# Impact of sodium citrate ingestion during recovery after strenuous exercise in the heat on heart rate variability: A randomized, crossover study

**DOI:** 10.14814/phy2.15280

**Published:** 2022-05-04

**Authors:** Janis Fiedler, Zane Šmite, Silva Suvi, Saima Timpmann, Martin Mooses, Luule Medijainen, Eve Unt, Vahur Ööpik

**Affiliations:** ^1^ Institute of Sports and Sports Science Karlsruhe Institute of Technology Karlsruhe Germany; ^2^ Faculty of Biology Department of Human and Animal Physiology University of Latvia Riga Latvia; ^3^ Institute of Sport Sciences and Physiotherapy University of Tartu Tartu Estonia; ^4^ Department of Sports, Medicine, and Rehabilitation Institute of Clinical Medicine University of Tartu Tartu Estonia; ^5^ Sports Medicine and Rehabilitation Clinic Tartu University Hospital Tartu Estonia

**Keywords:** dehydration, endurance athlete, exercise‐heat stress, plasma volume

## Abstract

Changes in hydration status influence plasma volume (PV) which is associated with post‐exercise parasympathetic reactivation. The present study hypothesized that, after dehydrating cycling exercise in the heat (DE), stimulation of PV expansion with sodium citrate (CIT) supplementation would promote heart rate variability (HRV) recovery in endurance‐trained men. Twelve participants lost 4% of body mass during DE. During subsequent 16‐h recovery, participants consumed water ad libitum (CIT =5.5‐L, PLC =5.1‐L) and ate prescribed food supplemented with CIT or placebo in a randomized, double‐blind, crossover manner. Relative changes in PV were assessed across DE and 16‐h recovery. HRV was analyzed before and 16 h after DE in three conditions for altogether four 5‐min periods: supine in a thermoneutral environment, supine in the heat (32°C, 46% relative humidity; 2 periods), and standing in the heat. A larger expansion of PV across 16‐h recovery occurred in CIT compared to placebo trial (*p* < 0.0001). However, no between‐trial differences appeared in HRV parameters (lnRMSSD, lnSDNN, lnLF/HF) in any 5‐min period analyzed before or 16 h after DE (in all cases *p* > 0.05). Increases in HR (*p* < 0.001) and lnLF/HF (*p* = 0.005) and decreases in lnRMSSD (*p* < 0.001) and lnSDNN (*p* < 0.001) occurred following DE in both trials. Larger PV expansion induced by CIT supplementation after DE does not improve recovery of HRV at rest and has no influence on HRV responsiveness in endurance‐trained men.

## Graphical abstract

Enhanced plasma volume expansion by sodium citrate supplementation after strenuous exercise did not improve the recovery of any HRV indices at rest. Additionally, 16 h are not a sufficient time for the recovery of HRV and HR measures after strenuous exercise in the heat.

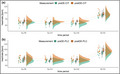

## 1 INTRODUCTION

Training and competing in road cycling are associated with high physical loads with limited recovery periods between consecutive activities (Jeukendrup et al., 2000; Padilla et al., 2000). Cycling events often take place in hot climates, which further increases the physiological demands placed on athletes (Cheuvront et al., 2010; Nybo et al., 2014). Appropriate fatigue and recovery monitoring tools are needed to properly dose training loads in different environments and to assess an athlete's level of readiness to compete. Exercise causes immediate parasympathetic inhibition with subsequent post‐exercise recovery. Therefore, monitoring post‐exercise parasympathetic nervous system (PNS) reactivation can be one tool to evaluate autonomic control during the recovery of cardiovascular function (Schaun & Del Vecchio, 2018; Stanley et al., 2013). Heart rate variability (HRV) analysis, which includes parameters that are used as a surrogate to assess the activity of the autonomic nervous system (ANS), is a promising non‐invasive method (Shaffer & Ginsberg, 2017), that has been employed for training monitoring and athlete's recovery status evaluation (Chrismas et al., 2019; Javaloyes et al., 2018; Schmitt et al., 2018). In particular, daily HRV‐guided training programs were shown to improve performance enhancement of peak power output and 40‐min time‐trial in cyclists (Javaloyes et al., 2018) and to prevent overreaching in skiers (Schmitt et al., 2018).

HRV is most commonly assessed in a supine position over a short period to assess the changes in autonomic modulation in the training process (Buchheit, 2014). However, as ANS activity is influenced by multiple factors (Buchheit, 2014; Fatisson et al., 2016; Parsons et al., 1992), HRV measurements in a single, steady‐state condition have their limitations. To overcome these limitations and to improve the sensitivity of HRV measurements, it has been proposed that researchers use different physiological provocations, for example, position change, to better identify changes in autonomic control (Howorka et al., 2010; Parsons et al., 1992; Schmitt et al., 2015; Schneider et al., 2019). Furthermore, thermoregulation is another function highly coordinated by ANS and a greater activation and stronger response of the sympathetic nervous system (SNS) to high ambient temperature have been associated with a better ability to cope with heat stress (Carrillo et al., 2016). While exercising in the heat, hyperthermia, and dehydration are the two major factors that influence heart rate (HR) and HRV and impair performance (Achten & Jeukendrup, 2003; Ebert et al., 2007; González‐Alonso et al., 1997; Hillman et al., 2011). Dehydration reduces parasympathetic activity and increases sympathetic activity after exercise in the heat which is reflected in various HRV parameters (Carter et al., 2005; Castro‐Sepulveda et al., 2014; Severeyn et al., 2016). Even if exercising in temperate environments, hydration status has been shown to impact the recovery of HRV measures (Vanderlei et al., 2015). Changes in hydration status influence plasma volume (PV) and an increase in PV has been shown to be associated with improvement in post‐exercise parasympathetic reactivation (Buchheit et al., 2009).

Athletes can use various fluid replacement strategies to induce PV expansion, including increasing sodium intake through salty meals, isotonic drinks, or specific supplements (Racinais et al., 2015). This may accelerate post‐exercise recovery, better maintain overall fluid and electrolyte balance and help to prevent the development of overreaching (insufficient recovery between exercises) (Shirreffs & Sawka, 2011). Moreno et al. (2013) demonstrated that hydrating with an isotonic solution throughout exercise and during post‐exercise recovery induced significant changes in cardiac autonomic modulation, promoting faster recovery of linear HRV indices. Sodium citrate (CIT) supplementation has been shown to lead to increased water retention and acute PV expansion after rapid weight loss in wrestlers and following dehydrating exercise in endurance athletes (Aedma et al., 2015; Timpmann et al., 2012; Vaher et al., 2015). Thus, given the results of previous research (Aedma et al., 2015; Buchheit et al., 2009; Moreno et al., 2013; Timpmann et al., 2012; Vaher et al., 2015), it seems likely that CIT supplementation after dehydrating exercise may influence post‐exercise parasympathetic reactivation which may be reflected in HRV indices. To the best of our knowledge, this possibility has never been controlled.

Therefore, the aim of the present study was to test the hypothesis that, after dehydrating exercise in the heat, stimulation of PV expansion with CIT supplementation would promote HRV recovery in endurance‐trained men.

## 2 MATERIALS AND METHODS

### 2.1 Participants

This work is based on HRV data that was collected in the framework of a complex study carried out on 20 endurance‐trained young men (Suvi et al., 2018, 2019) and the protocol of which was approved by the Research Ethics Committee of the University of Tartu (protocol 244/T‐16, January 19, 2015). However, HRV data from eight participants were excluded from the analysis due to syncopal episodes that occurred when they rose from a supine position to a vertical position in the heat (*n* = 3), or due to too many artefacts and measurement errors in the recordings (*n* = 5). Therefore, data of 12 men (mean ± SD; age, 30.8 ± 5.2 years; height, 1.81 ± 0.05 m; BM, 80.3 ± 9.6 kg, peak oxygen uptake (VO_2peak_), 55.9 ± 5.5 ml·kg^−1^·min^−1^) were used. Participants were non‐smokers, and their endurance training experience and routine training volume were 9.1 ± 7.3 years, and 9.8 ± 4.9 h per week, respectively. None of the participants had a history of heat illness nor had they traveled to a warm climate at least 2 months before the study. All participants gave their written informed consent to participate in this study after receiving detailed information about all the procedures.

### 2.2 Study design

This was a randomized, double‐blind, counterbalanced, crossover study with 7 days between CIT and placebo (PLC) trials. The study consisted of preparatory and main phases and was carried out at the exercise physiology laboratory of the University of Tartu in the winter–spring period (February–April) and in autumn (October–December) in 2015.

In the preparatory phase, the health and training status of potential participants was assessed using a questionnaire, they also underwent a medical examination, including resting electrocardiography. During subsequent visits of the eligible participants to the laboratory, they practiced breathing in the rhythm given by the metronome, their anthropometric data were collected and VO_2peak_ was measured in temperate environmental conditions (21–22°C, relative humidity (RH) 50%–51%). These procedures and equipment used are described in detail in our previous paper (Suvi et al., 2018).

**FIGURE 1:**
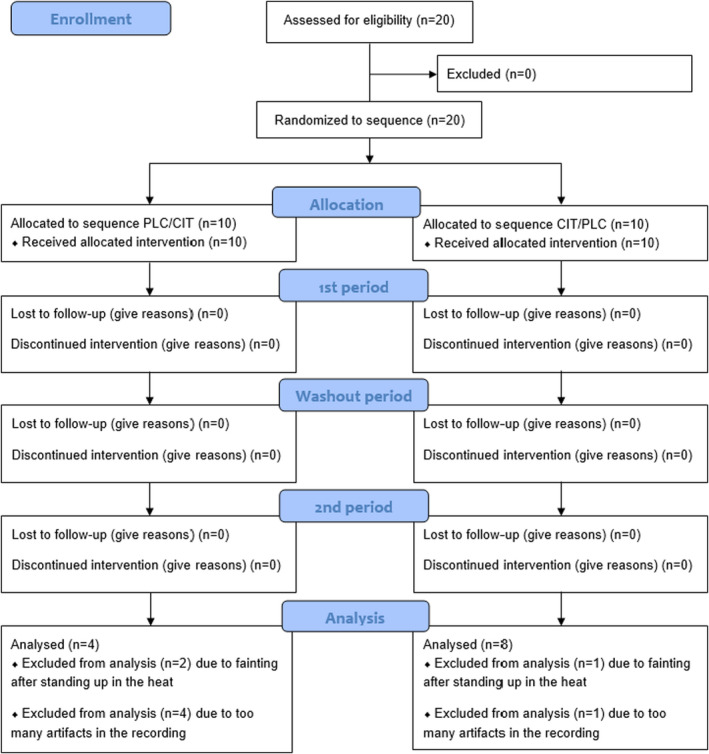
CONSORT flow diagram for crossover‐studies. Displayed is the randomized allocation to the groups placebo (PLC) and sodium citrate (CIT)

In the afternoon of the first day of the main phase of the study, well rested participants arrived at the laboratory. They voided, their nude body mass was measured to the nearest 0.001 kg using an electronic scale (CH3G‐150I Combics; Sartorius AG), and they were fitted with an HR transmitter strap and monitor. Then their HR was recorded under three conditions: (I) supine position at normal room temperature (21–22°C, RH 50%–51%); (II) supine position in the heat (32°C, RH 46%); (III) standing position in the heat (32°C, RH 46%) (Figure 1a).

**FIGURE 2:**
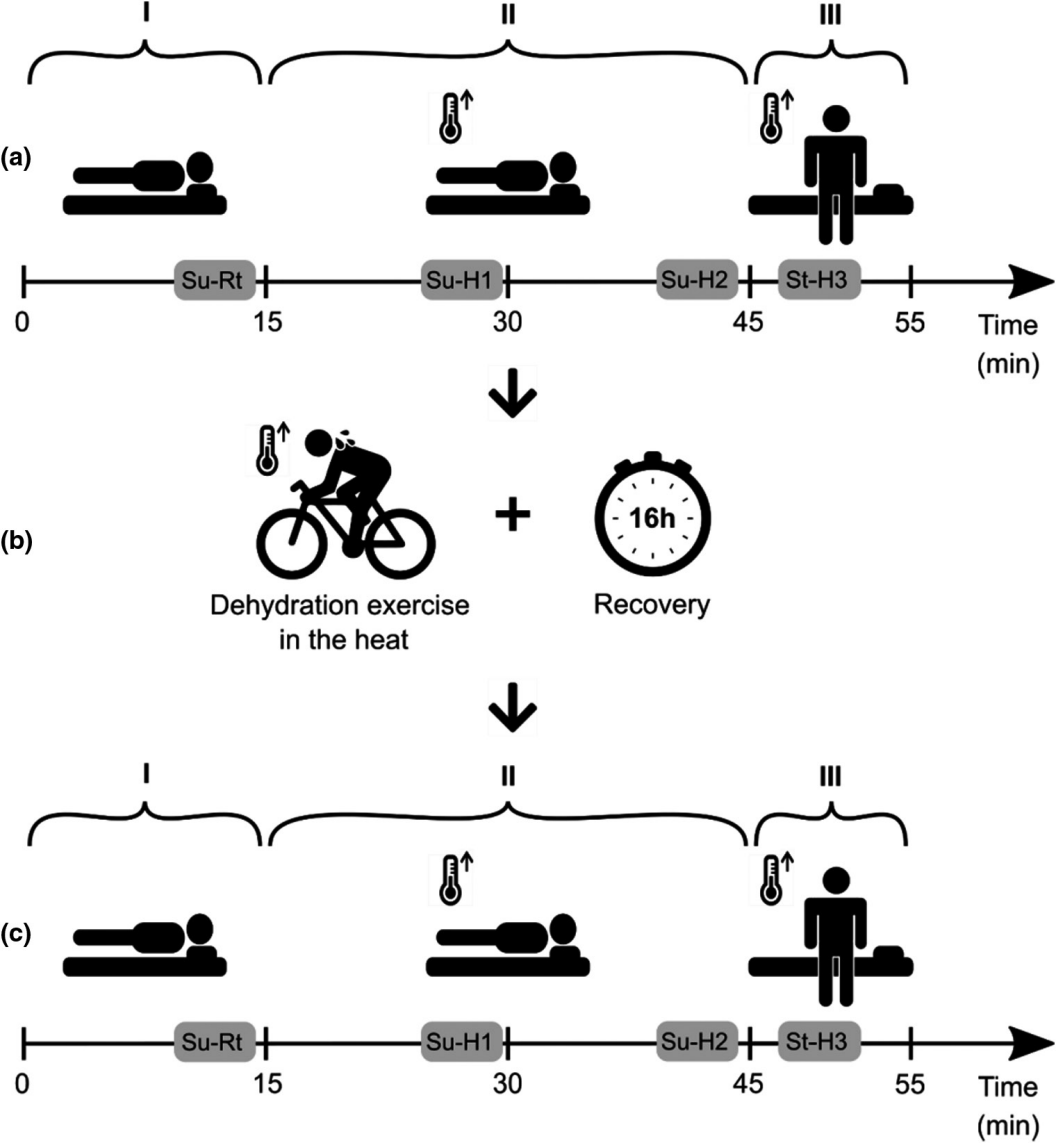
Conditions of HR recording and recorded HR time periods used for HRV analysis. HR was recorded under three conditions (I, II, III) and four recorded HR time periods (Su‐Rt, Su‐H1, Su‐H2, St‐H3) were selected for HRV analysis. These procedures were carried out before (a) and after (c) dehydrating exercise in the heat that was followed by a 16‐h recovery under normal environmental conditions (b)

Specifically, at normal room temperature participants spent 15 min in a supine position on a massage table (Su‐Rt). Next, they entered the climatic chamber (Design Environmental Ltd.) and took a supine position on a gym mat in the heat for 30 min (Su‐H1 and Su‐H2). Finally, still in the heat, they stood up and were standing for 10 min (Su‐H3). A total of four recorded HR time periods (Su‐Rt, Su‐H1, Su‐H2; St‐H3), 5 min each, were selected for HRV analysis (Figure 2a). A 5‐min time frame was set as the most stable part (no visual artefacts after correction) around the desired time (±2 min) with at least 30 s before the condition change to avoid flawed data. In detail: To minimize the influence of biphasic heart rate after position change (Hynynen et al., 2011), the first analyzed 5‐min period (Su‐Rt) started 6 min before the end of condition I. The second period (Su‐H1) was selected from the middle of condition II and started after 10 min of laying down. The third period (Su‐H2) started 6 min before the end of condition II, and the fourth period (St‐H3) started after 2 min of standing in condition III.

Following HR registration procedures, participants sat on a chair in the heat for 20 min prior to donating venous blood samples. Then, still in the heat, they moved to a Cyclus 2 ergometer (RBM Elektronik‐Automation GmbH) and completed dehydrating exercise in the heat (DE) (Figure 2b). During DE, participants exercised at 50%–60% of their individual VO_2peak_ until approximately 4% body mass loss was achieved. A more detailed description of DE is given in Suvi et al. (2018). A 16‐h recovery period under normal environmental conditions started immediately after DE (Figure 2b). During the recovery period, participants drank bottled water ad libitum, ate prescribed dinner and breakfast, and ingested gelatine capsules containing CIT (600 mg kg^−1^) or PLC (sucrose) in a randomized, double‐blind, crossover manner with 7 days between the two trials. A detailed description of the prescribed diet (it has been ensured that the diet prior to PLC and CIT was the same for each participant), as well as that of the procedure of CIT and PLC supplementation, is given in Suvi et al. (2018).

On the next morning, after the recovery period, participants came back to the laboratory and the HR recording and venous blood sampling procedures described above were repeated in the same order (Figure 2c). Thus, HR was recorded and HRV analyzed under three conditions (I, II, and III) twice: before DE (preDE) and following 16‐h recovery after DE (postDE).

### 2.3 HR interval recording and HRV data processing

Participants’ HR was recorded with an HR monitor Polar RS800CX with a one millisecond resolution (Polar Electro Oy). During HR recording, participants breathed 12 times per minute following the rhythm of the metronome. Controlled breathing rhythm is important to maintain stable ANS function during HR registration for later HRV analysis (Aubert et al., 2003). Recorded HR signals were retrieved from Polar Protrainer 5 software (Polar Electro Oy) and imported to the Kubios HRV Standard 3.0.2 software (Kubios Oy). The data was corrected with a manually applied filter and visually controlled to ensure that movement and other artefacts are removed. This resulted in a maximum correction of 3.19%.

The following parameters were used for complete case analysis of the data: Standard deviation of the time elapsed between two successive R‐waves (SDNN), root mean square of the sum of the squares of differences between adjacent R‐R intervals (RMSSD), low‐frequency/high‐frequency ratio from the FFT spectrum (LF/HF) and heart rate (HR).

### 2.4 Blood sampling and analyses, and calculation of relative change in plasma volume

Venous blood samples were collected from the antecubital vein preDE, directly after the exercise and after 16‐h recovery postDE into sterile serum Vacutainer tubes as well as into Vacutainer tubes containing ethylenediaminetetraacetic acid. Participants were sitting for 20 min in the heat preDE and postDE, while the blood samples were taken immediately after cycling while the participants were still on the cycle ergometer during both PLC and CIT. The blood samples were used for the measurement of hematocrit and hemoglobin concentration using a blood analyzer Celltac MEK‐6108K (Nihon Kohden). Blood was analyzed in duplicate without a correction for trapped plasma. The values obtained were used for the calculation of relative changes in PV (Dill & Costill, 1974). Additionally, blood collected into serum Vacutainer tubes was allowed to clot for 10 min at room temperature and was then centrifuged (Eppendorf 5804R, Eppendorf AG, Hamburg, Germany) for 10 min at 2700 *g* at 4°C. The separated serum was then used for the assessment of serum osmolality (SOsm).

### 2.5 Additional parameters for hydration status

Hydration status was assessed based on urine specific gravity (USG), SOsm, body mass, and water retention. Urine samples were collected from the participants before each DE. USG and SOsm were measured by means of a digital clinical refractometer (PDX‐CL; VeeGee Scientific Inc.) and freezing point depression osmometer Model 3250 (Advanced Instruments Inc.), respectively. Water retention during the 16‐h recovery period was calculated as difference between the volumes of water consumed and urine passed. Body mass changes were calculated on the basis of values measured before and after DE.

### 2.6 Statistical analysis

The raincloud plots have been created using RStudio (R Core Team, 2020) and the ggplot2 package (Wickam, 2016), following the instructions of Allan and colleagues (Allen et al., 2019).

SPSS 23 was used for the statistical calculations. Previous to the statistical calculations the data of the parameters SDNN, RMSSD, and LF/HF were log normal transformed to reduce skewness. The normal distribution was calculated using the Kolmogorov‐Smirnov test.

Because of the cross‐over study design, the impact of possible sequencing effects was calculated by performing an independent *t*‐test between the sum scores (preDE PLC + preDE CIT and postDE PLC + postDE CIT) of the groups in addition to a sufficient washout period (Wellek & Blettner, 2012).

Three‐way analysis of variance (ANOVA) (measurement (preDE‐postDE) × trial (PLC‐CIT) × time period (Su‐Rt, Su‐H1, Su‐H2, St‐H3)) for repeated measurements was used to compare the differences within and between the trials for RMSSD, SDNN, LF/HF ratio, and HR. If significant main effects for Time (×4) were found, the Bonferroni post hoc test was used to locate the differences between the means and control for multiple testing.

If Mauchly test of sphericity was significant and thus indicating that sphericity could not be assumed, the Huynh–Feldt correction has been applied when epsilon was >0.75 and when epsilon was <0.75 or nothing has been known about sphericity, the Greenhouse–Geisser correction was applied and adjusted values are displayed in the results section (Girden, 1992).

Two‐way (trial × time (during DE – during Recovery)) repeated‐measures ANOVA was used to evaluate the differences within and between the trials for PV, body mass, SOsm, and USG. Additionally, student's *t*‐test was used to analyze differences for water intake, urine, and water retention during the 16‐h recovery period.

## 3 RESULTS

### 3.1 Sequencing and carry‐over effects

Carry‐over effects were minimized due to a sufficient time sequence between the two trials (7 days). A potential sequencing effect, analyzed by using an independent *t*‐test, showed a significant difference between the sequence groups for baseline HR (CIT/PLC =118.76 ± 13.41 bpm, CIT/PLC =138.42 ± 11.55 bpm, *p* = 0.032) postDE. No further significant sequencing effect for any other time period and parameter were observed.

### 3.2 Main results

**FIGURE 3:**
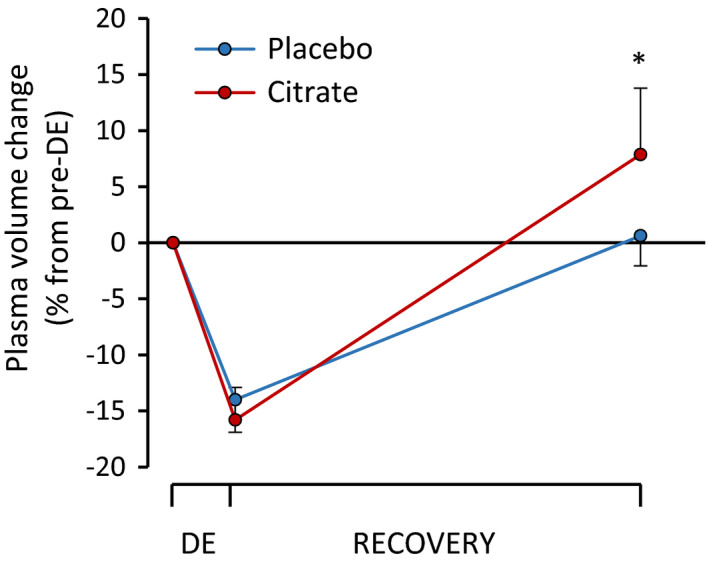
Plasma volume changes for the placebo and the citrate trials. Displayed is the plasma volume change relative to preDE (before dehydrating exercise) of 12 subjects (*y*‐axis) during dehydrating exercise in the heat (DE; *x*‐axis) and subsequent 16‐h recovery period (Recovery; *x*‐axis). Asterix marks a significant between‐trial difference (*p* < 0.05)

For PV changes, repeated measures ANOVA showed significant main effects of trial (*F* = 5.94, *p* = 0.033) and time (*F* = 200.03, *p* < 0.0001) and significant trial by time interaction (*F* = 18.68, *p* < 0.0001), indicating that participants had a smaller PV after DE, which recovered to (PLC) or above (CIT) the starting value (0) in both trials (Figure 3).

Table 1 shows the descriptive results for all HRV parameters while Table 2 shows the results regarding hydration status. Furthermore, water intake differed significantly (*p* = 0.018) between PLC (5099 ± 742 ml) and CIT (5493 ± 720 ml) while urine did not show a significant difference (*p* = 0.067) between PLC (1613 ± 642 ml) and CIT (1312 ± 314). Finally, water retention was significantly different (*p* > 0.001) between PLC (3486 ± 463 ml) and CIT (4181 ± 655 ml).

**TABLE 1:** Displayed are the log normal transferred means (and standard deviations) of 12 subjects before and after DE with the use of CIT and PLC for recovery for the parameters RMSSD, SDNN, and LF/HF ratio as well as the absolute HR values

Parameter	preDE/postDE	CIT/PLC	Su‐Rt	Su‐H1	Su‐H2	St‐H3
LnRMSSD	preDE	CIT	3.61 (0.50)	3.69 (0.51)	3.74 (0.52)	2.52 (0.62)
		PLC	3.57 (0.40)	3.76 (0.31)	3.93 (0.40)	2.67 (0.65)
	postDE	CIT	3.21 (0.49)	3.23 (0.60)	3.17 (0.44)	1.93 (0.49)
		PLC	3.27 (0.51)	3.29 (0.51)	3.50 (0.53)	2.00 (0.56)
LnSDNN	preDE	CIT	3.63 (0.36)	3.76 (0.41)	3.82 (0.40)	3.37 (0.47)
		PLC	3.60 (0.30)	3.80 (0.27)	3.99 (0.31)	3.43 (0.53)
	postDE	CIT	3.42 (0.41)	3.38 (0.49)	3.47 (0.39)	2.85 (0.42)
		PLC	3.35 (0.42)	3.50 (0.41)	3.71 (0.45)	2.91 (0.50)
lnLF/HF	preDE	CIT	0.02 (0.99)	0.64 (0.91)	0.52 (1.17)	2.25 (0.68)
		PLC	0.27 (0.83)	0.57 (0.91)	0.80 (0.98)	2.39 (0.72)
	postDE	CIT	0.63 (1.03)	0.82 (1.11)	0.97 (0.75)	2.54 (0.58)
		PLC	0.20 (0.95)	0.94 (1.20)	1.24 (1.03)	2.42 (0.64)
HR	preDE	CIT	58.34 (7.82)	56.73 (6.18)	56.09 (5.26)	90.94 (9.85)
		PLC	59.17 (7.52)	56.13 (4.96)	55.90 (5.73)	87.89 (10.94)
	postDE	CIT	67.79 (7.17)	64.67 (6.19)	66.87 (5.61)	96.48 (10.15)
		PLC	66.12 (8.89)	64.80 (5.75)	63.35 (5.96)	97.51 (9.34)

Abbreviations: CIT, sodium citrate trial; HR, heartrate; LF/HF, low frequency/high frequency ratio from the FFT spectrum; ln, log‐normal transformation; PLC, placebo trial; postDE, following 16‐h recovery after dehydrating exercise in the heat; preDE, before dehydration exercise in the heat; RMSSD, root mean square of the sum of the squares of differences between adjacent R‐R intervals; SDNN, standard deviation of R‐R intervals; St‐H3, standing position, 5 min periods of HR recordings selected for HRV analysis; Su‐H1, supine position, after 10 min in the heat; Su‐H2, supine position, after 25 min in the heat; Su‐Rt, supine position at room temperature.

**TABLE 2:** Displayed are the means (and standard deviations) of 12 subjects before DE, after DE, and after 16‐hour recovery with the use of CIT and PLC for the parameters body mass, SOsm, and urine specific gravity

Variable	Group	preDE	after DE	postDE
Body mass (kg)	PLC	80.58 ± 10.00	77.76 ± 9.76^a^	80.55 ± 9.96^b^
	CIT	80.26 ± 9.73	77.44 ± 9.52^a^	81.04 ± 10.03^a^, ^b^, ^c^
SOsm (mOsmol kg^−1^)	PLC	290.9 ± 3.7	299.5 ± 4.1^a^	291.8 ± 3.5^b^
	CIT	290.2 ± 3.7	301.5 ± 3.1^a^	295.1 ± 3.3^a^, ^b^, ^c^
Urine specific gravity	PLC	1.0105 ± 0.0100	–	1.0104 ± 0.0086
	CIT	1.0099 ± 0.0060	–	1.0102 ± 0.0074

Note

Significantly different (*p* < 0.05).

Abbreviations: after DE, directly after dehydration exercise in the heat, postDE, following 16‐h recovery after dehydrating exercise in the heat; CIT, sodium citrate trial; PLC, placebo trial; preDE, before dehydration exercise in the heat; SOsm, serum osmolality.

^a^ From before DH.

^b^ From after DH.

^c^ From PLC.

The three‐way ANOVA (measurement (preDE‐postDE) × trial (PLC‐CIT) × time period (Su‐Rt, Su‐H1, Su‐H2, St‐H3)) for repeated measurements showed no significant effects for the factor trial in any HRV indices or HR.

Significant main effects were found on the factor measurement for lnRMSSD (*F*(1, 11) = 83.18, *p* < 0.001, η²*p* = 0.88), lnSDNN (*F*(1, 11) = 64.78, *p* < 0.001, η²*p* = 0.86), lnLF/HF (*F*(1, 11) = 12.49, *p* = 0.005, η²*p* = 0.53) and HR (*F*(1, 11) = 62.96, *p* < 0.001, η²*p* = 0.85). This indicates lower values for lnRMSSD and lnSDNN, and higher values vor lnLF/HF and HR after DE compared to before DE.

The factor time period showed significant effects for lnRMSSD (*F*(1.45, 15.92) = 54.81, *p* < 0.001, η²*p* = 0.83), lnSDNN (*F*(1.54, 16.92) = 15.96, *p* < 0.001, η²*p* = 0.59), lnLF/HF (*F*(3, 33) = 67.36, *p* < 0.001, η²*p* = 0.86) and HR (*F*(1.37, 15.08) = 129.76, *p* < 0.001, η²*p* = 0.92). The post‐hoc Bonferroni analyses indicated that for lnRMSSD and HR only St‐H3 differed significantly from Su‐Rt, Su‐H1, and SuH2, for lnSDNN all but the difference between Su‐Rt and Su‐H1 were significant and for LF/HF all but the difference between Su‐H1 and SuH2 differed significantly. The post‐hoc Bonferroni analyses indicate, that standing up in the heat led to reduced lnRMSSD and lnSDNN values, while lnLF/HF and HR were higher. Additionally, some more inconsistent differences were found between the laying conditions.

**FIGURE 4:**
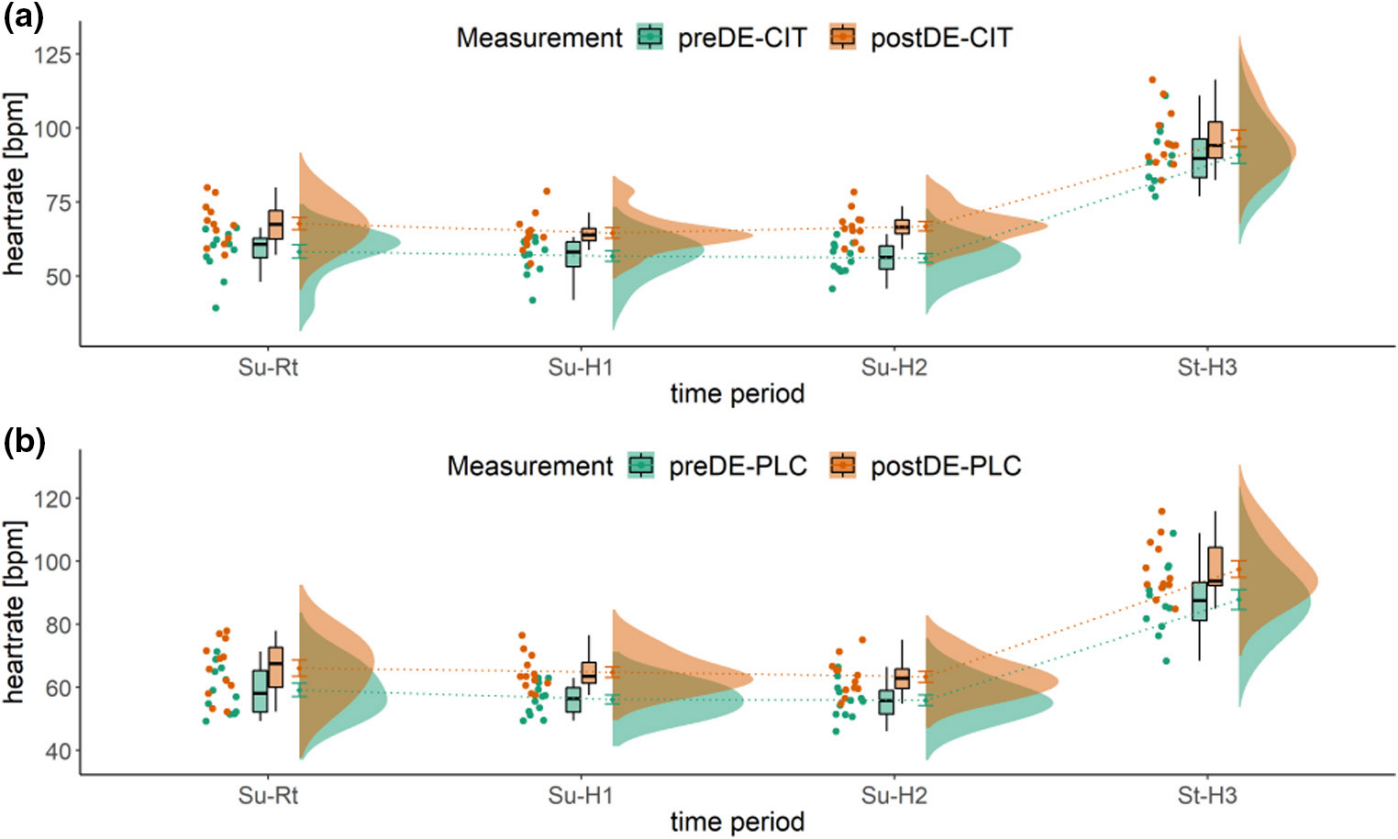
Interaction effect of measurement*trial*time period for the parameter heartrate. Displayed is the mean heartrate (y‐axis) of 12 subjects during 4 time periods (x‐axis) before dehydration exercise in the heat (preDE) and following 16‐h recovery after dehydrating exercise in the heat (postDE) with (a) sodium citrate (CIT) or (b) placebo (PLC) supplementation during recovery. Su‐Rt, supine position at room temperature; Su‐H1, supine position, after 10 min in the heat; Su‐H2, supine position, after 25 min in the heat; St‐H3, standing position, 5‐min periods of HR recordings selected for HRV analysis

HR also showed an interaction effect for time period*trial*measurement (*F*(1.71, 18.81) = 5.456, *p* = 0.017, η²*p* = 0.33) (see Figure 4).

## 4 DISCUSSION

The aim of this study was to test the hypothesis that, after dehydrating exercise in the heat, stimulation of PV expansion by CIT supplementation would promote a better recovery of HRV in endurance‐trained men. Results of this study demonstrate significantly larger PV expansion in CIT compared to PLC trial during the 16‐h recovery period after DE which is caused by a higher water retention (i.e. water intake vs. urine loss during 16‐h recovery) indicated by body mass, and serum osmolality changes with no change in urine specific gravity. However, contrary to our hypothesis, no between‐trial differences in HRV occurred. Specifically, the increased PV expansion caused by CIT supplementation did not affect either the HRV indices at rest or the HRV's responsiveness. On the other hand, in both CIT and PLC trials lnRMSSD and lnSDNN were significantly lower on the day after DE while HR and lnLF/HF were significantly higher. These findings indicate that 16‐h recovery after DE in the heat causing ~4% dehydration was inadequate for full recovery of HRV despite being at least fully rehydrated.

Previously it has been demonstrated that better hydration status promotes faster PNS reactivation post exercise (Carter et al., 2005; Moreno et al., 2013; Vanderlei et al., 2015) and it is thought that change in PV is an important regulator of parasympathetic reactivation via arterial‐baroreflex stimulation between one to 48 h following exercise (Stanley et al., 2013). In our study, despite that sodium citrate caused PV expansion, it did not lead to any change in HRV indices at rest. One possible explanation for this is that HRV parameters have been confounded so much by the exercise in the heat that improved PV expansion could not alter HRV indices on the following day. Compared to exercise in temperate conditions, exercise in heat induces a greater change in cardiovascular function (Achten & Jeukendrup, 2003; González‐Alonso et al., 1997; Nybo et al., 2014). Thus, restoring the cardiac and hemodynamic homeostasis takes longer time which also contributes to the slower recovery of HRV (Kenny & McGinn, 2017). In addition, even though PV change after rehydration in the CIT group was larger than in the PLC group, the PLC group did also recover to their baseline value. This indicates that even if a rapid fluid replacement can be advantageous in situations when recovery time between exercise sessions is limited (Moreno et al., 2013), longer recovery periods allow for appropriate rehydration just by drinking water ad libitum.

In contrast to our findings, Buchheit et al. (2009) reported that exercise induced PV expansion is positively associated with improved vagal‐related HRV indices based on post‐exercise supine measurement. Additionally, after a period of increased training load improved or non‐changed vagal‐related HRV indices have been reported despite increased fatigue (Buchheit, 2014; Buchheit et al., 2013; Hedelin et al., 2000). These training periods also led to PV expansion (Buchheit et al., 2013; Hedelin et al., 2000), so it has been speculated that the discrepancy between HRV and the state of fatigue can be associated with exercise induced PV expansion (Buchheit, 2014). However, in agreement with our results, later studies failed to demonstrate any relationship between the changes in PV and HRV after acute high intensity interval exercise sessions (Cipryan et al., 2016) or through a sauna‐based heat acclimation (Stanley et al., 2015). Thus, the utility of HRV measures to evaluate PV changes remains controversial. It seems that in a situation where the previous exercise has induced high physiological stress and during recovery athletes have managed to fully rehydrate, HRV is not sensitive enough to detect greater PV expansion.

Interpreting the results from a cardiovascular stress perspective shows that subjects were not fully recovered. Previous research showed that if overall exercise load is low then the activity of PNS can return to baseline within one day (Schaun & Del Vecchio, 2018; Stanley et al., 2013) but after exhaustive high intensity exercise it can take 48 h or more for vagal related HRV indices to return to pre‐exercise levels (Fazackerley et al., 2019; Stanley et al., 2013) which is confirmed by this study. It is also worth considering that there is a greater vagal withdrawal and increase of SNS activity when exercise is done under hot conditions (Brenner et al., 1997), and fluid loss amplifies these effects even more (Macartney et al., 2020). Thus, it is not surprising that 16‐h recovery along with rehydration was not a sufficient time period for HRV to return to pre‐exercise level after ~4% DE exercise in the heat in this study. However, additional characterization of inflammation, oxidative stress biomarkers (Bellafiore et al., 2021; Cooper et al., 2015; Hendrix et al., 2020), and psychological variables (Heidari et al., 2018) in the future studies may help to explain other contributing factors to HRV recovery in the hours to days following exercise in the heat.

In order to identify changes in autonomic control better, we used HRV measurements from a pre‐defined test protocol that included heat exposure and body position change which provoke the activation of SNS and withdrawal of PNS (Howorka et al., 2010; Schmitt et al., 2015). As there was no significant measurement by time interaction for any HRV indices, it seems that the current test protocol did not improve the sensitivity of the HRV analysis. Thus, from a practical perspective, it would be more feasible to use short‐term HRV measurements at rest, rather than through a complex and time‐consuming test. Even though a three‐way interaction effect for HR was found by this study it has to be noted that a significant sequencing effect for this parameter was present and therefore limits the interpretation.

Looking at the results for passive heating, it did not change vagal‐HRV indices (RMSSD) but HRV indices that are related to both PNS and SNS activity (SDNN, LF/HF ratio) increased. Therefore, thermoregulation due to mild passive heating for 30 min is associated with changes in autonomic nervous system activity. Other studies, where more substantial passive heating approaches have been used, have demonstrated parasympathetic withdrawal (Banjar et al., 2000; Crandall & Wilson, 2015; Laukkanen et al., 2019) and increased activation of SNS (Banjar et al., 2000; Carrillo et al., 2016). However, due to different heating approaches, comparison with these studies should be made with caution.

### 4.1 Limitations

The first limitation is the relatively small number of participants in this study with a dropout rate of 40%. However, it is in the nature of HRV analysis that measurement errors and artefacts occur and have to be controlled. Additionally, it has to be noted that the use of crossover study design while controlling for sequencing effects and having a sufficient wash‐out period requires fewer participants than parallel‐group trials (Wellek & Blettner, 2012).

Another limitation of this study is the use of mild heat stress and that the skin and core temperature changes were not measured parallel with HR recordings. Other studies in relatively mild environmental conditions have demonstrated that 30 min of heat stress (~37°C, 75% RH) influences HRV (Yamamoto et al., 2007). Additionally, passive exposure to heat stress (~33°C, 22% RH) for 7 days can provide strong enough stimuli to induce some cardiovascular and thermoregulatory adaptations (Pallubinsky et al., 2017). However, in both of these studies, one of the variables (ambient condition or exposure duration) that determines the rise in body heat storage was more intense than in the current protocol. Since athletes are more probable to engage with the milder heat stress condition, future studies should determine the duration of mild heat stress which has the potential to influence HRV. Here, measuring the skin and core temperature of the subjects during the trial would be needed in order to quantify how much heat is accumulating in the body.

The third limitation is that we did not have a baseline measurement over 7 days which is recommended for more precise data (Buchheit, 2014). In our study, we included an extended baseline measure including four measuring time points under different conditions instead and the results were consistent throughout the two conditions which indicates the stability of the measurements.

The fourth limitation regards the hypohydration which occurred due to the CIT consumption in the intervention group. As water intake was not restricted for the participants during the 16‐h recovery period, this was not considered by our a priori hypothesis. Hypohydration in participants of the PLC group might have resulted in different results.

Finally, the absence of HRV data directly after DE without a recovery period, and the absence of PV measurements after each HRV measurement period limits the interpretation of the true effect of the rehydration procedure.

## 5 CONCLUSION

In conclusion, our findings show that CIT supplementation‐induced enhanced PV expansion during 16‐h recovery from dehydrating exercise in the heat does not improve the recovery of any HRV indices at rest and has no influence on HRV responsiveness in endurance‐trained men.

## CONFLICTS OF INTEREST

The authors have no conflicts of interest to declare that are relevant to the content of this article.

## AUTHORS’ CONTRIBUTIONS

SS and VÖ were involved in the planning of the study design and SS, ST, MM, LM, and EU were in charge of conducting the experiment. JF and ZŠ processed the data and conducted the calculations. Interpretation of the data, and conducting of the first draft of the manuscript was done by JF and ZŠ with valuable comments from SS, VÖ, and ST. All authors read and approved the final version of the manuscript.

## CONSENT TO PARTICIPATE

All participants gave their written informed consent to participate in this study after detailed information about all the procedures.

## CONSENT FOR PUBLICATION

All authors read and approved the final manuscript for publication.

## ETHICS APPROVAL

The study was approved by the Research Ethics Committee of the University of Tartu (protocol 244/T‐16, January 19, 2015).
